# Mutational analysis of BRCA1 and BRCA2 genes in women with familial breast cancer from different regions of Colombia

**DOI:** 10.1186/s13053-019-0120-x

**Published:** 2019-07-15

**Authors:** Carolina Cortés, Ana Lucía Rivera, David Trochez, Melissa Solarte, Daniela Gómez, Laura Cifuentes, Guillermo Barreto

**Affiliations:** 1 Human Molecular Genetics Lab, Department of Biology, Universidad del Valle, Calle 13 No. 100-00 AA, 25360 Cali, Colombia; 2 Faculty of Sciences, Universidad Nacional Sede Manizales, Carrera 27 # 64-60, Manizales, Colombia; 3 Health Sciences, Universidad Cooperativa de Colombia Nariño, Calle 18 No. 47-150, Pasto, Colombia

**Keywords:** Breast cancer, BRCA1, BRCA2, Germline mutations, Familial cancer

## Abstract

**Purpose:**

The main risk factor for familial breast cancer is the presence of mutations in BRCA1 and BRCA2 genes. The prevalence of mutations in these genes is heterogeneous and varies according to geographical origin of studied families. In Colombia mutations in these genes have been mainly studied on patients from Andean region. Bogotá and Medellin presented its own battery of mutations. This study aims to identify mutations in BRCA1–2 genes in women with familial breast cancer from different regions of Colombia.

**Methods:**

One hundred four families with a history of breast cancer were sampled in different regions of Colombia, and the BRCA1 gene and exon 11 of the BRCA2 gene were sequenced. To predict the possible effects of sequence alterations found in protein function, different bioinformatics tools were used.

**Results:**

A total of 33 variants were found; 18 in BRCA1 and 15 in BRCA2, of which 15 are unique variants of Colombia. In silico analysis established that alterations p.Thr790Ala, p.Arg959Lys and p.Glu1345Lys in the BRCA1 gene and variants p.Leu771Phe, p.Asn818Lys, p.Val859Ser*22 and p.Lys1032Ile in the BRCA2 gene are considered likely pathogenic. Both the mutations as the variants of unknown clinical significance, in their great majority, presented a specific region distribution and they were different from those reported in previous studies.

**Conclusions:**

In this study we report the BRCA1 and BRCA2 spectrum of mutations and their distribution by regions in Colombia. Our results may help to design a diagnostic test including recurrent mutations for screening high risk to breast cancer families in Colombia.

## Background

Breast cancer is the most common malignancy and the second leading cause of cancer death affecting women worldwide [1]. In Colombia, this disease is the second-most frequently diagnosed malignancy, representing the leading cause of death in women according to statistics from the National Cancer Institute [2].

Breast cancer is a multifactorial disease that involves a complex combination of genetic and environmental factors. A positive family history of breast cancer increases the risk of developing this disease; this neoplasm is twice as common in women with affected first-degree relatives [3, 4]. It is estimated that most cases of this cancer are spontaneous; only 15 to 20% of cases are familial and associated with germline mutations in genes involved in the detection and repair of DNA damage [5].

In 1994 and 1995, the first genes associated with susceptibility to breast cancer, BRCA1 (OMIM # 113705) and BRCA2 (OMIM # 600185), were identified. It has been learned that both genes play an important role in genome integrity and maintaining chromosomal stability because they perform multiple functions related to tumor suppressor activity, including roles in repairing DNA, cell cycle control, chromatin remodeling, apoptosis and transcriptional regulation. Therefore, a loss of function in BRCA1/2 genes results in an altered sequence or aberrant expression of these proteins that, can trigger many cellular defects, including genomic instability and cell transformation that leads to tumorigenesis [6]. BRCA1/2 genes account for approximately 25% of all cases of familial breast cancer. Families with mutations in these genes usually have several members affected; carriers of mutations in BRCA1 have a 70–80% chance of developing the disease, and their risk of developing ovarian cancer is 40% [7]. Similarly, carriers of mutations in BRCA2 have a 40–84% risk for breast cancer and an 11–27% risk for ovarian cancer [8].

Considering the high rate of morbidity and mortality of breast cancer in the Colombian population, it is important to make an early diagnosis of mutational status of genes associated with breast cancer predisposition such as BRCA1 and BRCA2 with the purpose of establishing genetic tests to reduce the risk of developing this disease and to facilitate the monitoring of asymptomatic carriers. In Colombia, there are 5 prior studies on breast cancer. The first of these, performed on 57 patients from Bogota, found two mutations in BRCA1 and three mutations in BRCA2. The authors concluded that these mutations are founders of the Colombian population [9, 10]. Other two studies with women from Medellin and other Colombian cities reported to carry different mutations to founder from Bogotá [11, 12]. A fourth study compared two methods for mutation detection (complete direct sequencing of BRCA1 and BRCA2 genes on 30% of the sample vs partial sequencing of these genes on 70% of patients for detection of six known mutations) on 853 Colombian women detected 107 mutations, of which 69 were located in BRCA1 and 38 in BRCA2 [13]. Finally, Torres et al. (2017) [14] utilizing different methods of mutation detection studied the prevalence of BRCA1/2 mutations in Colombian familial and unselected breast cancer patients from Andean region mainly (Bogotá, Neiva and Villavicencio) observing the presence of two novel founder mutations in BRCA2 (1991 del 4 and ex 1–4 del in BRCA2) additionally to a novel large BRCA2 deletion (ex1-14del), identified in 0.9% of the screened families.

These studies differ in terms of the results obtained as each analyzed city reported its own battery of mutations; this can be explained by the size of the samples evaluated, variability and sensitivity of the techniques used to identify mutations, ethnicity (amerindian, afrodescendent or European origin patients) and geographic distribution. This study utilizing only the direct sequencing methodology of BRCA1 gene and exon 11 of BRCA2 gene aimed to identify mutations that increase the familial susceptibility to breast cancer in families from different regions of Colombia.

## Methods

A total of 104 unrelated families from different regions of Colombia: Pacific region (27), Caribbean region (24), Coffee Region (29), Andean region (8) and Eastern Region (16), who had family histories of breast and/or ovarian were analyzed; their identities were obtained from different institutions and organizations that had records of patients diagnosed with breast cancer and that served as intermediaries for contacted patients. The patients were selected with at least one of the following criteria that classify them as high risk families 1. have two relatives in the first degree affected with breast and / or ovarian cancer, and at least one of them diagnosed before 41 years of age for breast cancer or with ovarian cancer at any age. 2. At least three relatives in the first or second degree of the same family line, affected with breast and / or ovarian cancer at any age 3. At least one case of breast cancer diagnosed before 35 years of age, preferably bilateral and 4. At least one case of ovarian cancer diagnosed before 30 years of age, preferably bilateral [15].

Patients selected and their families were informed about the purpose of the investigation, and those who chose to participate voluntarily signed the informed consent. The characteristics of the patients included in the study are reported in (Table 1). This research was approved by the ethic board of the Universidad del Valle (Internal code 082 act No. 004).

**Table 1:** Patients characteristics, *N* = 104

Characteristics	Number (%)
Family history of breast or ovarian cancer	
No	39 (37,5%)
Yes	65 (62,5%)
Age at diagnosis	
> 45	49 (47,12%)
< 45	55 (52,88%)
Bilateral breast cancer	
No	99 (95,19%)
Yes	5 (4,81%)
Type	
Invasive Ductal Carcinoma	92 (88,46%)
Invasive Lobular Carcinoma	12 (11,54%)

### DNA extraction and quantification

All participants provided a sample of 5 ml of peripheral blood in vacutainer tubes with EDTA. DNA extraction was performed from 1 ml of peripheral blood by the “salting-out” extraction method [16]. The DNA obtained was quantified by spectrophotometer Nanodrop ND 2.000.

### Amplification and sequencing

To determine the presence of mutations in patients, direct sequencing was used. Initially, for each patient, all exons (24) of BRCA1 and exon 11, (representing 43% of the size) of the BRCA2 gene were amplified by PCR. BRCA1 amplification was performed using primers for each gene coding region as reported by Barker [17]. For amplification of exon 11 of BRCA2, primers reported by the *Breast Cancer Information Core (BIC)* were used, dividing this exon into 15 fragments with an average size of 410 bp.

The amplification program consisted of 2 min at 94 °C followed by 30 cycles of the following: 45 s at 94 °C, 60 s at 60 °C, 60 s at 72 °C and 10 s at 72 °C. This, was carried out on Applied Biosystems Thermocycler Veriti™. Each PCR reaction was performed in a final volume of 25 μl, with 50 ng of DNA template, 1 U/μL of Taq polymerase, 0.4 μM of primers, 0.1 Mm of dNTPs, 1.25 Mm of MgCl2, reaction buffer 1X and ultrapure water to the final volume. The amplified products were sequenced on an Applied Biosystem ABI Genetic Analyzer 3130 (HITACHI).

Sequencing electropherograms were analyzed using ChromasPro version 1.5. The presence and location of abnormal sequence were obtained by sequence alignment using BLAST (Basic Local Alignment Search Tool) and taking as reference sequences for BRCA1 (DNA NG_005905.1 and RNA: U14680.1) and to *BRCA2* (DNA NG_012772.3 and RNA: MN_000059.3). To name the detected variants, we used the nomenclature adopted by the Human Genetic Variation Society (HGVS).

To predict the possible effects of sequence alterations found in protein function, the following programs were run: SIFT (Sorting Intolerant From Tolerant), Polyphen2 (Prediction of Functional Effects of Human nsSNPs), MutPred 1.2 (Mutation Prediction), PROVEAN (Protein Variation Effect Analyzer), PANTHER (Protein Analysis Through Evolutionary Relationships), ALIGN GVGD, SNP & GO and Alamut Visual 2 [18–25].

## Results

When we analyzed the BRCA1 gene and exon 11 of BRCA2 gene of the 104 families by direct sequencing 33 sequence alterations were found, 18 in BRCA1 gene and 15 in BRCA2. Eighteen of these variants were polymorphic and shared with other populations in Latin America (11 in BRCA1 and 7 in BRCA2), and 15 sequence alterations are first reported in this study (7 in BRCA1 and 8 in BRCA2) (Table 2).

**Table 2:** Found variants in the BRCA1 gene and exon 11 of the BRCA2 gene

Gene	Fragment	Number of patients	Identified alteration	A.A	Classification of alteration	Reported in databases	Clinical significance –BIC
BRCA1	7	1	c.412 C > A	p.Leu138Ile	Non-synonymous (M)	NO	NA
	11	2	c.4186-22G > A	NA	Intronic	NO	NA
	11	2	c.2077 G > A	p.Asp693Asn	Non-synonymous (M)	YES	Neutral
	11	2	c.2079 G > A	p.Asp693Asp	Synonymous	YES	Unknow
	11	30	c.2082 C > T	p. Ser694Ser	Synonymous	YES	Neutral
	11	2	c.2146 T > A	p.Ser716Arg	Non-synonymous (M)	NO	NA
	11	13	c.2311 T > C	p.Leu771Leu	Synonymous	YES	Neutral
	11	2	c.2368 A > G	p.Thr790Ala	Non-synonymous (M)	YES	Unknow
	11	64	c.2612 C > T	p.Pro871Leu	Non-synonymous (M)	YES	Neutral
	11	2	c.2876G > A	p.Arg959Lys	Non-synonymous (M)	NO	NA
	11	3	c.3083G > A	p.Arg1028His	Non-synonymous (M)	YES	Unknow
	11	27	c.3113A > G	p.Glu1038Gly	Non-synonymous (M)	YES	Neutral
	11	1	c.3506A > C	p.Asp1169Ala	Non-synonymous (M)	NO	NA
	11	20	c.3548A > G	p.Lys1183Arg	Non-synonymous (M)	YES	Neutral
	11	1	c.3978 T > G	p.His1326Gln	Non-synonymous (M)	NO	NA
	11	4	c.4033G > A	p.Glu1345Lys	Non-synonymous (M)	NO	NA
	13	8	c.4308 T > C	P.Ser1436Ser	Synonymous	YES	Unknow
	16	17	c.4837A > G	P.Ser1613Gly	Non-synonymous (M)	YES	Neutral
BRCA2	11.0	1	c.1909-37dup	NA	Intronic (IVS)	NO	NA
	11.0	9	c.1909-33delA	NA	Intronic (IVS)	NO	NA
	11.1	1	c.2147A > G	p.Gln716Arg	Non-synonymous (M)	NO	NA
	11.1	2	c.2229 T > C	p.His743His	Synonymous (Syn)	YES	Neutral
	11.1	1	c.2313A > T	p.Leu771Phe	Non-synonymous (M)	NO	NA
	11.1	18	c.2386G > A	p.Asp796Asn	Non-synonymous (M)	NO	NA
	11.1	1	C.2454 T > A	p.Asn818Lys	Non-synonymous (M)	NO	NA
	11.2	1	c.2574_2575insA	p.Val859Ser*22	Non-sense (N)	NO	NA
	11.3	7	c.2971A > G	p.Asn991Asp	Non-synonymous (M)	YES	Neutral
	11.3	1	c.3095ª > T	p.Lys1032Ile	Non-synonymous (M)	NO	NA
	11.4	23	c.3396 T > G	p.Lys1132Lys	Synonymous (Syn)	YES	Neutral
	11.5	8	c.3807 T > C	p.Val1269Val	Synonymous (Syn)	YES	Neutral
	11.7	80	c.4563A > G	p.Leu1521Leu	Synonymous (Syn)	YES	Neutral
	11.13	75	c.6513C > G	p.Val2171Val	Synonymous (Syn)	YES	Neutral
	11.14	3	c.6841 + 80_6841 + 83delTTAA	NA	Intronic (IVS)	YES	Neutral

M = misense, N = nonsense

Of all alterations found in BRCA1 and BRCA2 genes, 57.6% were not synonymous changes; 27.3% were sequence alterations that do not lead to a change of amino acid in the protein, 12.12% were sequence alterations located in noncoding regions (intron regions) and 3% of alterations led to a stop codon.

After analyzing the alterations in silico, it was determined that seven (21.2%) of alterations identified in this study are probably deleterious (Table 3); 3 in BRCA 1 and 4 in BRCA2 gene. These pathogenic mutations were identified in two regions, in the pacific region were p.Thr790Ala, p.Arg959Lys, p.Glu1345Lys, and p.Val859Ser*22, and in the coffee region were p.Leu771Phe, p.Asn818Lys y p.Lys1032Ile.

**Table 3:** Predictive analysis using bioinformatics packages for new mutations of unknown clinical significance for the BRCA1 gene and exon 11 of the BRCA2 gene

					Prediction Aminotacidic Change						Splice Signal Detection	
Gene	Exon	cDNA	Protein Change	Align-GVGD	PANTHER (SubSPEC)	PROVEAN (Score)	SIFT (Score)	PolyPhen-2 (Prob.)	MutPred (Prob.)	SNP&GO (RI, EA)	5’or 3′ score modification (% variation)	
BRCA1	**7**	c.412C > A	p.Leu138Ile	Prob. Not Deleterious (C0)	Neutral	Neutral (−0.167)	Tolerated	Benign (0.201)	Prob. Not Deleterious	Disease (4,0.714)		
					(−0.156)		−0.056		(0.233)			
	**I11**	c.4186-22G > A	NA	NA	NA	NA	NA	NA	NA	NA	Not alter splicing site	
	**11**	c.2146 T > A	p.Ser716Arg	Prob. Deleterious	Neutral	Neutral (−1.673)	Tolerated	Benign (0.002)	Prob. Not Deleterious	Neutral (3,0.354)		
				(C65)	(−0.109)		(0.537)		(0.288)			
	**11**	**c.2368 A > G**	**p.Thr790Ala**	**Prob. Deleterious**	**Neutral (−2)**	**Deleterious (−3.873)**	**Tolerated (0.059)**	**Prob. Damaging (0.926)**	**Prob. Deleterious**	**Disease (6,0.775)**		
				**(C55)**					**(0.419)**			
	**11**	**c.2876G > A**	**p.Arg959Lys**	**Prob. Deleterious**	**Neutral (−1)**	**Neutral (− 2.126)**	**Tolerated (0.081)**	**Prob. Damaging (0.0.81)**	**Prob. Deleterious**	**Disease (8,0.878)**		
				**(C25)**					**(0.448)**			
	**11**	c.3083G > A	p.Arg1028His	Prob. Deleterious	Neutral	Neutral (0.117)	Tolerated (0.304)	Benign (0)	Prob. Not Deleterious	Disease (4,0.723)		
				(C25)	(−2.906)				(0.391)			
	**11**	c.3506A > C	p.Asp1169Ala	Prob. Deleterious	Neutral	Neutral (2.020)	Damaging	Benign (0.077)	Prob. Not Deleterious	Disease (2,0.580)		
				(C 65)	(−1.960)		(0.036)		(0.267)			
	**11**	c.3978 T > G	p.His1326Gln	Prob. Deleterious	Neutral	Neutral (−1.689)	Damaging	Benign (0.294)	Prob. Not Deleterious	Disease (4,0.708)		
				(C15)	(−1.918		(0.013)		(0.177)			
	**11**	**c.4033G > A**	**p.Glu1345Lys**	**Prob. Deleterious**	**Neutral (−1.767)**	**Neutral (−0.754)**	**Damaging**	**Prob. Damaging (0.48)**	**Prob. Not Deleterious**	**Disease (6,0.799)**		
				**(C55)**			**(0.002)**		**(0.186)**			
BRCA2	I10	c.1909-37dup	NA	NA	NA	NA	NA	NA	NA	NA	MaxEnt	5.19 3.47 (−33.14%)
											HSF:	73.17 72.04 (−1.54%)
	I10	c.1909-33delA	NA	NA	NA	NA	NA	NA	NA	NA	GS:	6.5 7.0 (7.14%)
	11	c.2147A > G	p.Gln716Arg	Prob. Not Deleterious (C0)	Neutral	Neutral	Tolerated	Benign	Neutral	Disease		
					(−1,58)	(−1.79)	(−0,12)	(0.001)	(0.111)	(4, 0.703)		
	**11**	**c.2313A > T**	**p.Leu771Phe**	**Prob. Not Deleterious (C0)**	**Neutral**	**Deleterious**	**Damaging**	**Damaging**	**Neutral**	**Disease**		
					**(−2,078)**	**(−3,155)**	**(0.03)**	**1**	**(0.221)**	**(2, 0.588)**		
	**11**	c.2386G > A	p.Asp796Asn	Prob. Not Deleterious (C0)	Neutral	Neutral	Damaging	Benign	Neutral	Disease		
					(−1.5)	(−1.678)	(0.01)	(0.031)	(0.18)	(6, 0.779)		
	**11**	**c.2454 T > A**	**p.Asn818Lys**	**Prob. Not Deleterious (C0)**	**Neutral**	**Deleterious**	**Damaging**	**Benign**	**Neutral**	**Disease**		
					**(−1.1)**	**(−4.026)**	**(0.01)**	**(0.024)**	**(0.346)**	**(8, 0.916)**		
	**11**	**c.2574dup**	p.Val859Ser*22	**NA**	**NA**	**NA**	**Damaging**	**NA**	**NA**	**NA**		
	**11**	**c.3095A > T**	**p.Lys1032Ile**	**Prob. Not Deleterious (C0)**	**Neutral**	**Deleterious**	**Damaging**	**Prob. Damaging**	**Prob. Deleterious**	**Disease**		
					**(−2.6)**	**(−7.108)**	**0**	**(0.971)**	**(0.504)**	**(9, 0.929)**		

Entries in boldface indicate likely deleterious mutations

The most frequent variants (without clinical significance) in BRCA1 were p.Pro871Leu (observed in 61.5% of patients), p.Ser694Ser (28%), and p.Glu1038Gly (26%); for BRCA2, they were p.Leu1521Leu (81.7%), p.Val2171Val (72%) and p.Lys1132Lys (23%). The alterations reported as polymorphisms were specific by region, as follows: in the Pacific region were p.Asp693Asn and p.Asp796Asn; in the Caribbean region were p.Gln716Arg, p.Asp991Asn and c.1910-37dup and in the coffee region was c.1909-33delA. The other eight polymorphisms were shared between the regions analyzed (Table 4).

**Table 4:** Specific Mutations by Region

Region	Pathogenic Mutations		Polymorphisms	
	BRCA1	BRCA2	BRCA1	BRCA2
Pacific	p.Thr790Ala p.Arg959Lys p.Glu1345Lys	p.Val859Ser*22	p.Asp693Asn	p.Asp796Asn
Caribbean	NO	NO	NO	p.Gln716Arg p.Asn991Asp c.1910-37dup
Andean	NO	NO	NO	NO
Coffee	NO	p.Leu771Phe p.Asn818Lys p.Lys1032Ile	NO	c.1909-33delA
Eastern	NO	NO	NO	NO

*NO* not observed

## Discussion

Studies previous in Colombia had been realized utilizing different methods for mutations detection (PTT, SSCP, multigene kit panel, sequencing) in women with breast cancer mainly from Andean region [9–14]. In this study we utilized only the sequencing strategy (of BRCA1 gene and exon 11 of BRCA2 gene) and families with histories of breast and/or ovarian cancer from different regions (Pacific, Caribbean, Coffee, Andean and Eastern) from Colombia. In 104 families studied, we detected 33 sequence alterations. Eighteen (11 in BRCA1 and 7 in BRCA2 genes) are considered polymorphic by the consulted database and are shared by other Latin-American populations. The remaining 15 sequence alterations have not yet been reported (7 in BRCA1 and 8 in BRCA2). Seven of these (21.2%) variants were considered likely deleterious according to the bioinformatics program analysis (3 in BRCA1 and 4 in BRCA2), and 10 are probably neutral (6 in BRCA1 and 4 in BRCA2). The majority of polymorphism were specific by geographic region.

### In silico analysis

We used in silico programs to make predictions based on sequences conservation (SIFT, PROVEAN, PANTHER and SNP&GO) [18, 21, 22, 24], wild-type amino acid properties against the mutated, evolutionary information (Align-GVGD) [23], and the assessment of the structural stability of the protein (Polyphen2 and MutPred) [19, 20]. The use of all these programs and the combination of all their characteristics gives a high reliability to the prediction because they analyze these variants from different angles thereby increasing the preciseness of it. The results of in silico analysis give a preliminary view of the possible functional effects of the studied variants, and its predictions can establish the basis for additional experiments (functional and multifactorial analysis).

#### BRCA1 alterations

Nine of the alterations were assessed by bioinformatics tools resulting in 3 likely deleterious alterations. The first mutation, c.2368A > G, causes a change from threonine to alanine at 790 position (p.Thr790Ala). This specific mutation has been reported in the BIC database as an unknown clinically important variant in Afro-descendant women. Even though the breast cancer incidence in Afro-descendant women is less than in Caucasian women, a high percentage of Afro-descendant women are diagnosed at younger ages [26]. Coincidentally, the patient who harbored this alteration in the current study was an Afro-descendant woman diagnosed with breast cancer at the age of 35, whose familial history was unknown. It is said that this pathology presents a unique behavior in the clinical and biological development of Afro-descendant women. [27]. The contribution of the mutations in BRCA genes have not been well-assessed yet in Afro-descendant women; however, some studies reveal the existence of particular mutations in this specific ethnic group [28].

The second mutation, c.2876G > A, was found in the same region that produces a switch in position 959 for arginine to lysine (p.Arg959Lys). This alteration has not yet been reported in other countries. It was identified in a patient with unilateral breast cancer who had an extensive familial breast cancer history of 8 cases in addition to her own. This result might be due to the positive charge of the arginine and lysine located in the protein surfaces. These two amino acids play an important role in protein stability because they allow ionic interactions and hydrogen bonds [29]. Even though both amino acids work as basic residues, arginine residues provide a more stable structure to the protein than lysine. Additionally, the alteration of electrostatic interactions induced by the mutation of arginine to lysine affects protein folding, decreasing the production of a functional protein [30].

The third mutation, c.4033G > A, is reported for the first time. Such a base substitution transition type leads to a change in glutamic acid (negative charge) to a lysine (positive charge) at position 1,345 (p.Glu1345Lys), which is a change of an acid to a base. This alteration was identified in a clinical case whose familial history reported prostate cancer and a second breast cancer case. The p.Glu1345Lys mutation could obstruct the formation of the BRCA1-CBP/P300 complex, resulting in a loss of the suppressor function of tumors that BRCA1 possesses [31].

In short for BRCA1, according to these results, p.Thr790Ala, p.Arg959Lys and p.Glu1345Lys alterations are proposed as mutations with probable deleterious functions. Two of these, p.Arg959Lys and p.Glu1345Lys, have been observed for the first time in the Colombian population. These predictions are in accordance with the functional data since these mutations are located in regions of exon 11 of BRCA1 at the interface with proteins involved with chromosome stability, like RAD51 and P300/CBP.

#### BRCA2 alterations

Eight mutations were detected within exon 11 of BRCA2 gene. To assess these, bioinformatics tools were used to classify four of them as probably deleterious.

BRCA2 p.Val859Ser*22 leads to the generation of an early stop codon at position 881 of the BRCA2 protein (22 amino acids after mutation site). This protein in its natural state presents 3,418 amino acids; this mutation causes a 74% loss of the entire protein, approximately 2,537 amino acids. Consequently, several BRC repeats are lost in the central region. In addition, the entire carboxyl-terminal region is lost as well, including the binding domains of both single-stranded DNA (ssDNA), double-stranded DNA (dsDNA), and the nuclear localization signal of BRCA2. The analysis of this alteration by the SIFT program states that this truncated protein would suffer from the mRNA decay process mediated by non-sense sequences (mRNA Nonsense Mediated Decay). This process is a cell monitoring mechanism that detects mRNA with early termination codons and proceeds to degrade them. This process is very important because translated mRNA with early stop codons can produce truncated proteins with deleterious gain-of-function activity or dominant-negative protein activity [32].

Thanks to all these studies, we can conclude the p.Val859Ser*22 mutation is likely pathogenic. Beyond the evidence shown by the SIFT program, when a truncated mutation appears as a result in a genetic test it is considered highly probable as the actual cause of the disease [33].

The three remaining sequence alterations are base substitutions that generate missense mutations. Two of these, are located within the interaction site of BRCA2 and NPM1. This region includes all amino acids from 639 to 1,000 in the BRCA2 protein [34]. The programs indicating that the p.Leu771Phe mutation is probably deleterious are those that look at sequence conservation (SIFT, Polyphen 2 and PROVEAN). Moreover, they show that lysine is highly conserved at this site. As the amino acid change occurs in a conserved site, it is probable that it is a deleterious alteration.

The remaining alteration that the programs disagree on is p.Asn818Lys. Three of the programs predict that the sequence is conserved here, therefore, the change is deleterious and would affect the protein structure. We therefore conclude that this variant is probably deleterious.

Finally, the remaining missense alteration (p.Asn1032lys) is located within the first repeats of BRC (BRCA1 a.a. 1,003-1,036). As expected, this alteration is predicted in silico by several programs to be a deleterious alteration. BRCA1 repeats are some of the most conserved within mammal along with the BRCA2, 4, 7 and 8 repeats. As mentioned, BRC repeats have the function to link up to RAD51 monomers and transport them to the damage site inside the double-stranded chain to start the homologous recombination repair process. Carreira & Kowalczykowski [35] demonstrated that BRCA1, 2, 3 and 4 repeats, have the highest affinity for RAD51, take monomers to the damage site and bind RAD51 to the single-stranded DNA, allowing RPA protein displacement. Later, these 4 repeats keep the union between RAD51 and DNA, avoiding binding to double-stranded DNA and allowing RAD51 to start the process of invasion of the nonhomologous strand for repair by homologous recombination. Even though there are not as many BRC repeats in other organisms as in humans, most of them are necessary to BRCA2-RAD51 function in the repair of double-stranded DNA breaks. Therefore, mutations in only one BRC repetition could increase breast cancer risk.

### Population analysis

Considering the most of cancer predisposition genes are heterogeneous, it has seen that these genes have hundreds of sequence alterations that cause diseases and some of them have strong founder effects in certain populations, which are inherited and often remain restricted to specific geographic regions [36]. For Colombia, Torres et al. [9], established the prevalence of three founder mutations for this population “Profile Colombia”, however, the results found in this study suggest the existence of other different founder mutations (p.Thr790Ala, p.Arg959Lys, p. Glu1345Lys for BRCA1 and p.Leu771Phe, p.Asn818Lys, p.Val859Ser * 22, p.Lys1032Ile for BRCA2) that may be modulating the development of breast cancer in the country. These results infer that the spectrum of mutations differs completely from one region to another due to the specificity of the genetic structure present in Colombia. Therefore, it is necessary to perform additional studies that analyze large series of families with breast cancer in different geographical regions to accurately estimate the prevalence of mutations in these populations.

The alterations found in the sampled population are shared with Spain (c.4308 T > C y c.4837A > G) [37] and some Latin American populations, such as those from Peru (c.2082C > T, c.2311 T > C, c.2612C > T, c.3113A > G, c.3548A > G, c.4308 T > C, c.2229 T > C, c.2971A > G, c.3396 T > G, c.3807 T > C, c.4563A > G) [38], Venezuela (c.4308 T > C, c.4837A > G, c.2229 T > C, c.2971A > G, c.4563A > G, c.6513C > G) [39], Argentina (c.2077 G > A, c.2612 C > T, c.3113A > G c.3548A > G, c.2971A > G) [40, 41], Brazil (c.2082 C > T, c.2368A > G, c.2612 C > T, c.2229 T > C, c.3396 T > G, c.3807 T > C, c.4563A > G, c.6513C > G) [42, 43], Mexico (c.6841 + 80_6841 + 8 3delTTAA) [44] and Chile (c.2311 T > C, c.3113A > G, c.3548A > G, c.2971A > G, c.3396 T > G) [45, 46]; it is important to note, however, that not all studies sequenced the BRCA1 and BRCA2 genes. In addition, most of them used PTT; with this, it is not possible to obtain information about the presence of these polymorphisms in all Latin American populations that have been reported in genetic studies including these two genes. The polymorphisms found in the present study of the Colombian population were not comparable to the studies performed by Torres et al. [9, 10] because they did not analyze exon 11 of BRCA1 and BRCA2 with sequencing but instead used PTT. As a result, they reported only nonsense mutations for these two exons. In addition, the studies performed by Rodríguez et al. [47] with patients in Bogotá, Montería, Sucre, and Londoño et al. [11] with patients in Neiva and Medellin, were not comparable either because they assessed the Hispanic panel mutation in patients with either breast cancer or ovarian cancer not selected by familial history.

The clinically unimportant alterations found in the BRCA1 gene and exon 11 of the BRCA2 gene showed differences in their distribution in the evaluated regions. For the BRCA1 gene, the p.Asp693Asn alteration was present in the Pacific region and not shared with the rest of regions analyzed in the country. The p.Leu771Phe variant was detected in the Pacific, Atlantic and Andean regions. The p.Ser1613Gly alteration was identified in all regions except for the Andean and Eastern regions. The p.Ser694Ser, p.Pro871Leu, p.Glu1038Gly and p.Lys1183Arg alterations were shared in all regions evaluated. Two of the alterations found in exon 11 of BRCA2 gene were present in the Pacific region (p.Asp796Asn, p.Val859Ser*22). In the Atlantic coast region, only 3 of these alterations were present (p.Gln716Arg, p.Asp796Asn, c.1910-37dup). These two regions (Pacific and Atlantic) shared the p. Asp796Asn alteration. Finally, 4 alterations present in the Andean region were not shared with the rest of regions (p.Leu771Phe, p.Asn818Lys, p.Lys1032Ile, c.1910-33delA).

At present, thanks to the mtDNA (mitochondrial DNA) and the Y chromosome, we can infer that the gene pool of the Colombian population is the result of a complex process of mixing of three parental populations (Native American, European and African) [48]. The proportions of the parental populations fluctuate between every region in the country, making them diverse one from another. For instance, the Antioquia, Caldas, Quindío (Coffee region) and Santander (Eastern region) populations present a higher mix of European and Native American. On the other hand, the Bolívar, Magdalena, Cauca and Valle del Cauca present an equal mix of the three parental populations [48].

In contrast, given that Colombia has one of the most diverse populations in Latin America, and due to its great geographical heterogeneity and marked regional differentiation, the identification of mutations in a specific population could be due to historical reasons such as the reduction of a certain parental population during the colonization process and/or selective advantages of mutations [48]. Similarly, several mutations appear exclusively in one or few countries possibly due to the limited number of investigations made to date [36].

## Conclusions

The results obtained in this study allowed us to find 33 sequence alterations for the BRCA1/2 genes, of which 7 alterations are cataloged as probably pathogenic, findings that contribute to the knowledge of the mutational spectrum that may be modulating the development of this neoplasia in the Colombian population. However, to establish the role of these mutations, it is necessary to perform functional studies to elucidate their effect on the pathology. Both the mutations as the variants of unknown clinical significance, in their great majority, presented a specific region distribution and they were different from those reported in previous studies. Consequently, in order to establish a panel of Colombian mutations our results emphasize the need to expand the sample size in each region in order to be more representative of the whole country. Establishment of a panel of Colombian mutations will allow proposing preventive and adequate options for asymptomatic carriers.

## Data Availability

The datasets generated and/or analyzed during the current study are not publicly available due we do not have the authorization of the patients but are available from the corresponding author on reasonable request.
